# Successful recovery from severe hypertension in a patient with Leigh syndrome

**DOI:** 10.1016/j.ymgmr.2020.100684

**Published:** 2020-11-29

**Authors:** Yuto Arai, Kiyotaka Kosugiyama, Takuya Tamura, Sasagu Matsumoto, Akira Sudo, Hideaki Shiraishi, Cammack Ivor, Akira Ohtake, Kiyoshi Nagumo

**Affiliations:** a Department of Pediatrics, Teine-Keijinkai Hospital, Sapporo, Japan; b Department of Pediatrics, Hokkaido University School of Medicine, Sapporo, Japan; c Social Welfare Corporation Nire-no-kai Children's Clinic, Sapporo, Japan; d Department of Clinical Residency Training, Teine-Keijinkai Hospital, Sapporo, Japan; e Center for Intractable Diseases, Saitama Medical University Hospital, Saitama, Japan

**Keywords:** Leigh syndrome, Hypertension, Respiratory failure, Medulla oblongata, Nucleus tractus solitarii

## Abstract

Hypertension is a rare complication of Leigh Syndrome (LS), but prognosis of patients with hypertension is poor and its presence is indicative of the terminal stage of the disease. Herein, we report a four-year-old girl case diagnosed with LS at 15 months of age who subsequently developed severe hypertension and respiratory failure. Physical examination and laboratory findings did not indicate a secondary cause of hypertension. Her respiratory failure was treated with non-invasive ventilation and hypertension controlled with enalapril, furosemide and spironolactone. To our knowledge, this is the first case of a patient with LS recovering from severe hypertension.

## 1 Introduction

Leigh syndrome (LS) is a progressive neurodegenerative disorder presenting in infancy or early childhood. Clinical manifestations include psychomotor regression with loss of previously acquired motor and mental skills, associated with signs of basal ganglia and brainstem involvement [1]. Brain computed tomography and magnetic resonance imaging (MRI) show bilateral symmetrical necrotic lesions of the basal ganglia and brainstem [2]. In the majority of cases, dysfunction of the mitochondrial respiratory chain is responsible for the disease and LS is caused by either mitochondrial or nuclear gene mutations with large genetic heterogeneity [3]. Usually, patients with *SURF-1* deficiency have reduced activity in complex IV and relatively mild symptoms and better survival [4,5]. Hypertension is rarely associated with this condition, but based on previous reports, usually indicates the terminal phase of the illness [[6], [7], [8], [9]]. We report a case of LS with *SURF-1* mutation complicated by fulminant hypertension where the patient survived.

## 2 Case report

A four-year-old girl presented with respiratory distress.

She was born to healthy parents after a 37-week pregnancy and normal delivery with a birth weight of 2478 g. There was no family history of mitochondrial disease. Although she had full head control at 4 months, she was only able to roll over at 10 months and sit without support at 14 months. A diagnosis of LS was made at 15 months, when she was admitted to our hospital with an episode of decreased activity. Investigations showed increased lactate and pyruvic acid in the cerebrospinal fluid, and hyperintense lesions in the bilateral putamina, midbrain and medulla oblongata on T2-weighted images (T2WI). Muscle biopsy demonstrated type 2 fiber atrophy and moderate fiber size variation without lymphoid aggregates and no ragged-red fibers on modified Gomori trichrome. Respiratory chain analysis in the muscle showed complex IV residual activity was 7.7% of the control mean value relative to citrate synthase, while the activities of complexes I, II, III and citrate synthase were within the normal range. Sanger sequencing revealed a compound heterozygous mutation of *SURF-1* [c.826_827ins18 (p.Val276_Thr277ins6), c.743C > A (p.Ala248Asp)].

**Fig. 1:**
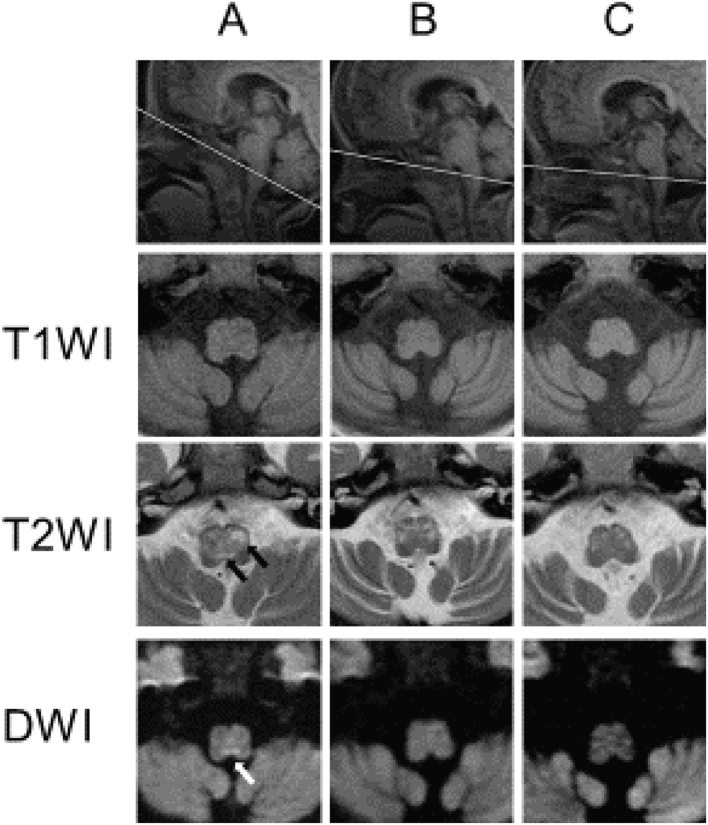
T2-weighted magnetic resonance images of the brain taken at the age of three years show increased signal intensity in the rostral and dorsal portion of medulla oblongata (black arrows) and diffusion weighted magnetic resonance images show high intensity signal in the dorsal part of medulla oblongata (white arrow) (A). T2-weighted MRI scans at the age of four years show slightly diminished high signal intensity of rostral portion of medulla oblongata and nearly complete disappearance of high intensity signal in the dorsal portion of the medulla oblongata (B). T2-weighted magnetic resonance images at the five years show more regression of the high intensity signals in the dorsal portion of medulla oblongata (C).

Despite best supportive cares, her motor and mental functions gradually deteriorated. She developed epilepsy and lost neck control and the ability to sit. She also developed dystonia and dysphagia and required nasogastric tube feeding and full assistance with activities of daily living. She was enrolled in a clinical trial of 5-Aminolevulinic acid (5-ALA) with sodium ferrous citrate (SFC) at two years of age. Follow-up MRI at the age of three showed new lesions in the rostral and dorsal portion of the medulla oblongata on T2WI (Fig. 1A). High intensity signal in the dorsal part of the medulla oblongata on diffusion weighted images (DWI) was also noted. However, neither hypertension nor any respiratory symptoms were noted at this point.

At four years of age, she had sudden episodes of sighing respiration and rapid gasping, lasting several seconds. She was also lethargic, but there was no weight change, flushing, or tremor. There were no signs of infection or sick contacts.

On physical examination, she was pale and drowsy. Her body temperature was 36.3 °C, oxygenation on room air was 99%, respiratory rate was 24/min with irregular sighing respirations. Her heart rate was 126/min and regular and her blood pressure was 150/100 mmHg. Pulmonary examination showed no crackle or wheeze. Cardiac examination revealed no murmur. Her abdomen was soft and distended without palpable masses with hypoactive bowel sounds. There was no abdominal bruit. Her skin was clammy with cool peripheries. There were no signs of excessive cortisol.

Serum electrolytes were within the normal range (sodium of 137 mmol/L, potassium of 4.5 mmol/L and chloride of 104 mmol/L). Renal function was also within normal limits (urea nitrogen of 15.9 mg/dL and creatinine of 0.15 mg/dL). We performed a capillary blood gas which showed respiratory acidosis (pH of 7.340, pCO2 of 46.3 mmHg, HCO3 of 24.3 mmol/L and base deficit of 1.3 mmol/L), lactate of 17 mg/dL and glucose of 108 mg/dL. Chest X-ray showed no abnormality. We were unable to identify the cause of her respiratory distress and hypertension, so she was admitted for monitoring and further investigation.

Her respiratory function gradually declined. Capillary blood gas analysis taken 4 four days after admission showed worsening respiratory acidosis (pH 7.310, pCO2 57.5 mmHg, HCO3 28.1 mmol/L). She stabilized with non-invasive ventilation (NIV) but the abnormal respiratory pattern persisted.

**Fig. 2:**
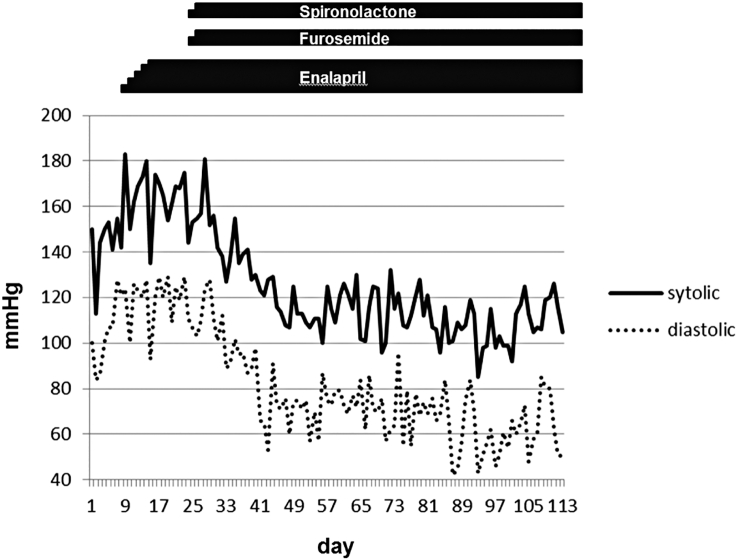
Daily peak blood pressures (systolic and diastolic) and initiation of antihypertensive medication. Her blood pressure decreased after furosemide and spironolactone were initiated.

Her blood pressure gradually increased to an average of 180/100 mmHg nine days after admission (Fig. 2). There were no findings suggesting organ damage and circulatory failure. We initiated treatment with oral enalapril on day nine which was titrated up to 0.35 mg/kg/d. We then added oral spironolactone at 2 mg/kg/d and furosemide at 1.5 mg/kg/d on day 26, and her blood pressure subsequently normalized. Her admission was complicated by aspiration pneumonia and after a long period of rehabilitation, she was finally discharged after 114 days with ongoing NIV. She was normotensive after discharge and antihypertensive therapies were gradually tapered.

Endocrine investigations were performed on day 11. Raised serum epinephrine levels of 1100 pg/mL (normal reference range 100–400 pg/mL) and norepinephrine levels of 1420 pg/mL (normal <70 pg/mL) and normal dopamine levels of 20 pg/mL (normal <30 pg/mL) were revealed. Serum steroids levels were also slightly raised (ACTH of 73.2 pg/mL, cortisol of 23.2 μg/dL). Thyroid function was normal (TSH of 0.51 μIU/mL and FT4 of 1.51 ng/dL). Abdominal ultrasound did not find any adrenal masses or renovascular stenosis.

An MRI obtained 70 days after admission demonstrated symmetrical high intensity signals of the rostral medulla oblongata on T2WI, which had slightly regressed compared to images obtained at three years of age (Fig. 1B). Signal intensity abnormalities of dorsal portion of the medulla oblongata on T2WI and DWI showed nearly complete disappearance.

Follow-up MRI obtained at the age of five years demonstrated more regression of the rostral medulla oblongata abnormalities on T2WI (Fig. 1C).

## 3 Discussion

Hypertension is uncommon as a presenting sign in mitochondrial respiratory chain disorders, but four cases of hypertensive crisis have been reported (Table. 1) [[6], [7], [8], [9]]. All of the cases had respiratory abnormalities, brain stem lesions involving the medulla oblongata, needed multiple antihypertensive agents and died within several months. Our patient was treated similarly, and we expected her to have a limited prognosis. However, she successfully recovered from the hypertensive crisis and we would like to propose three contributing factors.

**Table 1:** Cases of severe hypertension in LS patients.

Patient	1	2	3	4	5
	Pamphelett et al. [6]	Narita et al. [7]	Lohmeier et al. [8]	Brecht et al. [9]	Our Case
Age (years)	11	8	3	2	4
Maximum BP (mmHg)	180/130	200/140	200 (sys)	140/100	180/120
Respiratory abnormality	+	+	+	+	+
Brain stem involvement	+	+	+	+	+
Antihypertensive drugs	Propranolol Hydralazine Chlorothiazide	Propranolol Nifedipine	Propranolol Nifedipine Captopril	Propranolol Amlodipine Nifedipine Captopril	Enalapril Spironolactone Furosemide
Mechanical ventilation	−	−	+	+	+
Results	Died (day 7)	Died (day 9)	Died (day 30)	Died (day 44)	Alive
Cause of death	Unknown	Respiratory failure	Sepsis	Unknown	
Gene	Untested	Untested	Unidentified	*MT-ND5*	*SURF-1*

Firstly, the period of mechanical ventilation probably provided some respite to recover from the acute attack. Patient 2 of the reported cases was treated without a ventilator and died of respiratory failure relatively quickly [7]. On the other hand, patients 3 and 4 were treated with a ventilator and had longer survival [8,9]. It is also possible that her acute exacerbation was milder than previously reports [[1], [2], [3], [4], [5], [6], [7], [8], [9], [10], [11], [12], [13], [14], [15]] because she has a mutation in the *SURF-1* gene, which is associated with milder disease and better survival than those with classical gene mutation: *SCO2*, *SLC19A3* and *BOLA3* [4,5].

Secondly, it may be due to the choice of antihypertensive therapy. We used a previously unreported combination of antihypertensive medicine: enalapril, furosemide and spironolactone [[6], [7], [8], [9]]. Fig. 2 shows the reduction in blood pressure after initiating furosemide and spironolactone suggesting that diurectics were more effective in this case. On the other hand, enalapril is an angiotensin converting enzyme (Fig. 1, Fig. 2) (ACE) inhibitor that decreases the formation of angiotensin II. Recently there is a report that angiotensin II in the brain controls blood pressure by modulating sympathetic outflow to the periphery [10,11]. It is not known whether enalapril crosses the blood-brain barrier but it may have provided some additional benefit.

Thirdly, the patient's trial drug - 5-ALA with SFC could have contributed. 5-ALA is a heme precursor that acts as a protein-bound prosthetic group in mitochondrial respiratory chain complexes II, III, and IV, and cytochrome *c*, all of which are important in aerobic metabolism [12]. This drug has been reported to enhance heme production, leading to respiratory complex upregulation [12]. This clinical trial is research conducted by AO to investigate the efficacy and safety of 5-ALA with SFC administered to mitochondrial diseases centered on cranial nerve symptoms. However, since this clinical trial is still in progress, the efficacy is unproven.

The cause of our patient's hypertension is difficult to detect, but the nucleus tractus solitarii (NTS), located in the dorsal portion of the medulla oblongata, play an important role in adjusting blood pressure and maintaining eupnea [13]. We speculate that lesions in this area of the brain may have contributed to the severe hypertension and respiratory distress.

Our study has several limitations. Firstly, as brain MRI was performed 70 days after admission, it may not reflect the neurological abnormalities of the acute attack. However, Arii et al. reported that the timing of fatal respiratory failure with LS patients cannot be predicted from MRI findings [14]. Our patient already had bilateral lesions in the dorsal portion of the medulla oblongata possibly involving the NTS one year before admission (Fig. 1). Based on this, precise monitoring of respiratory function and blood pressure in LS patients particularly with brainstem lesions may be necessary. A second limitation was the difficulty in ruling out secondary hypertension. We attempted to evaluate for a secondary cause, but in particular pheochromocytoma was difficult to exclude. Her catecholamine levels were high but pheochromocytoma was thought to be highly unlikely as her hypertension subsided after discharge. Kvetnansky et al. reported that stress induces catecholamine enzymes, primarily through hypercortisolism [15]. Because she had elevated catecholamine and cortisol levels, it is likely that these are the result of a stress response to her acute exacerbation of LS symptom.

## 4 Conclusion

This concluded our report of a young girl with LS who managed to recover from fulminant hypertension. The cause of hypertension was unclear but probably neurogenic. Precise monitoring of blood pressure and respiratory pattern in LS patients is important as they are unpredictable and may indicate the terminal stage of the illness. To the best of our knowledge, this is the first report of a patient with LS who has recovered after the onset of severe hypertension. Further research is required to discover effective treatments for the hypertension seen in LS which may help to modify the natural course of this disease.

## Consent

Informed consent was obtained from the patient's parents for genetic studies and publication of this case report. The patient participated in the clinical trial with the consent of her parents and the Institutional Review Board (IRB) at the hospital.
